# Psychological stress and diastolic blood pressure in cardiology outpatients: a multicenter cross-sectional study (from the ABC2X-2026 study)

**DOI:** 10.3389/fcvm.2026.1865898

**Published:** 2026-06-17

**Authors:** Giuseppe Berton, Mattia Ludovico Dario, David Merotto, Rocco Cordiano, Elena Selvestrel, Silvia Rui, Heba Talat Mahmoud

**Affiliations:** 1 ABC Study Foundation—ETS, Conegliano, Italy; 2 Cardiology Outpatient Clinic, Conegliano General Hospital, Treviso, Italy; 3 Cardiology Outpatient Clinic, Adria General Hospital, Rovigo, Italy; 4 RSA Padre Pio Srl, Tarzo, Treviso, Italy

**Keywords:** cardiology outpatients, cardiovascular risk, coronary artery disease, diastolic blood pressure, psychological stress, psychosocial factors

## Abstract

**Background:**

Psychological stress is increasingly recognized as an independent cardiovascular risk factor, yet its hemodynamic correlates in routine cardiology practice remain little characterized. In particular, the clinical implications of distinguishing between ongoing and previous stress have been seldom explored.

**Methods:**

This multicenter cross-sectional study was conducted across three cardiology outpatient clinics in Northern Italy. Between 2019 and 2025, a total of 675 consecutive adult patients undergoing their first outpatient evaluation were enrolled. Psychological stress was assessed through a structured physician-guided clinical interview and categorized as no stress, present stress, or previous stress. Blood pressure (BP), heart rate (HR), clinical characteristics, and lifestyle factors were recorded during the visit. Multinomial logistic regressions were used to identify determinants of stress timing and stressors, while linear regression models were used to evaluate associations between stress (present and previous) and cardiovascular parameters.

**Results:**

Overall, 373 patients (55%) reported psychological stress: 264 (39%) had present and 109 (16%) previous stress. Present stress was independently associated with younger age, female sex, lower physical activity, and anxiolytic treatment, whereas previous stress was independently associated with post-COVID enrollment. In multivariable linear regression models, present stress was independently associated with higher diastolic blood pressure (DBP) (*β* = 3.0 ± 0.9 mmHg, *p* = 0.001), whereas previous stress was not. Neither present nor previous stress was significantly associated with systolic BP or HR. Interaction analyses showed that the association between present stress and DBP became more evident with increasing age (β = 0.15 ± 0.06 mmHg, *p* = 0.007) and was significantly much stronger in patients with coronary artery disease (CAD) (β = 5.9 ± 2.4 mmHg, *p* = 0.01).

**Conclusions:**

In cardiology outpatients, psychological stress is highly prevalent and shows distinct hemodynamic correlates according to its timing. Ongoing, but not previous, stress is independently associated with higher DBP, particularly among elderly, and patients with CAD. Routine assessment of psychological stress may improve cardiovascular risk stratification in outpatient practice.

## 1 Introduction

Psychological stress is a recognized cardiovascular risk factor, acting independently of other traditional predictors (1). The 2016 and 2021 ESC guidelines recommended its integration into individual risk assessment, as it significantly influences the development and progression of cardiovascular disease (1, 2). This clinical priority is further underscored by the 2025 ESC Clinical Consensus Statement, which advocates for systematic mental health screening and a multidisciplinary “*Psycho-Cardio Team*” approach (3). Evidence from the INTERHEART study, conducted across 52 countries, confirms that psychosocial factors are important independent predictors of myocardial infarction, regardless of geographic or ethnic background (4). Both acute and chronic stress have been shown to trigger autonomic imbalance through activation of the hypothalamic–pituitary–adrenal axis and sympathetic overactivity (5–7). This cascade increases catecholamine release, peripheral vascular resistance, and endothelial dysfunction, collectively impairing vascular reactivity (8, 9). While initially adaptive, prolonged exposure promotes systemic inflammation through activation of the neuro-cardiac axis and the release of pro-inflammatory cytokines, as well as maladaptive structural brain remodeling (6, 7, 10, 11). These mechanisms may compromise BP regulation and hemodynamic stability (8–10). However, the clinical expression of these stress-related alterations remains heterogeneous among studied populations, highlighting the need to better characterize individual risk modifiers. Chronic exposure to stressors may promote sustained hypertension through autonomic imbalance and sympathetic overactivity (12–15). Although meta-analytic evidence confirms a significant association between stress and BP elevation, findings regarding specific hemodynamic responses—such as systolic blood pressure (SBP) vs. DBP—remain inconsistent (16–20). Elevated heart rate (HR) is also considered a marker of cardiovascular risk in hypertensive states (21). Despite these insights, data on how these parameters interact in cardiology outpatient settings remain limited. Psychometric scales are rarely adopted in routine cardiology practice, where stress is often evaluated through physician-guided clinical interviews (22). The reliability of single-item direct measures to identify psychological burden has been well-established (23, 24). The clinical relevance of self-reported stress is further supported by long-term data from Kivimäki et al. (25). However, to the best of our knowledge, no studies have distinguished between ongoing (present) stress and stress experienced in the past but no longer active. This temporal distinction may be clinically relevant, as persistent stress exposure could lead to different hemodynamic effects compared with previous stress. Moreover, specific sources of stress, ranging from financial/work-related to health-related factors, may influence both the presence and the intensity of psychological stress (25–29). In this multicenter cross-sectional study of cardiology outpatients, we aimed to investigate the association between psychological stress and cardiovascular parameters assessed during routine clinical evaluation. We sought to distinguish present and previous stress, to explore predominant sources of stress (stressors), and assess potential interactions between stress, age, and coronary artery disease (CAD) (30–33).

## 2 Materials and methods

### 2.1 Study design and population

**Figure 1:**
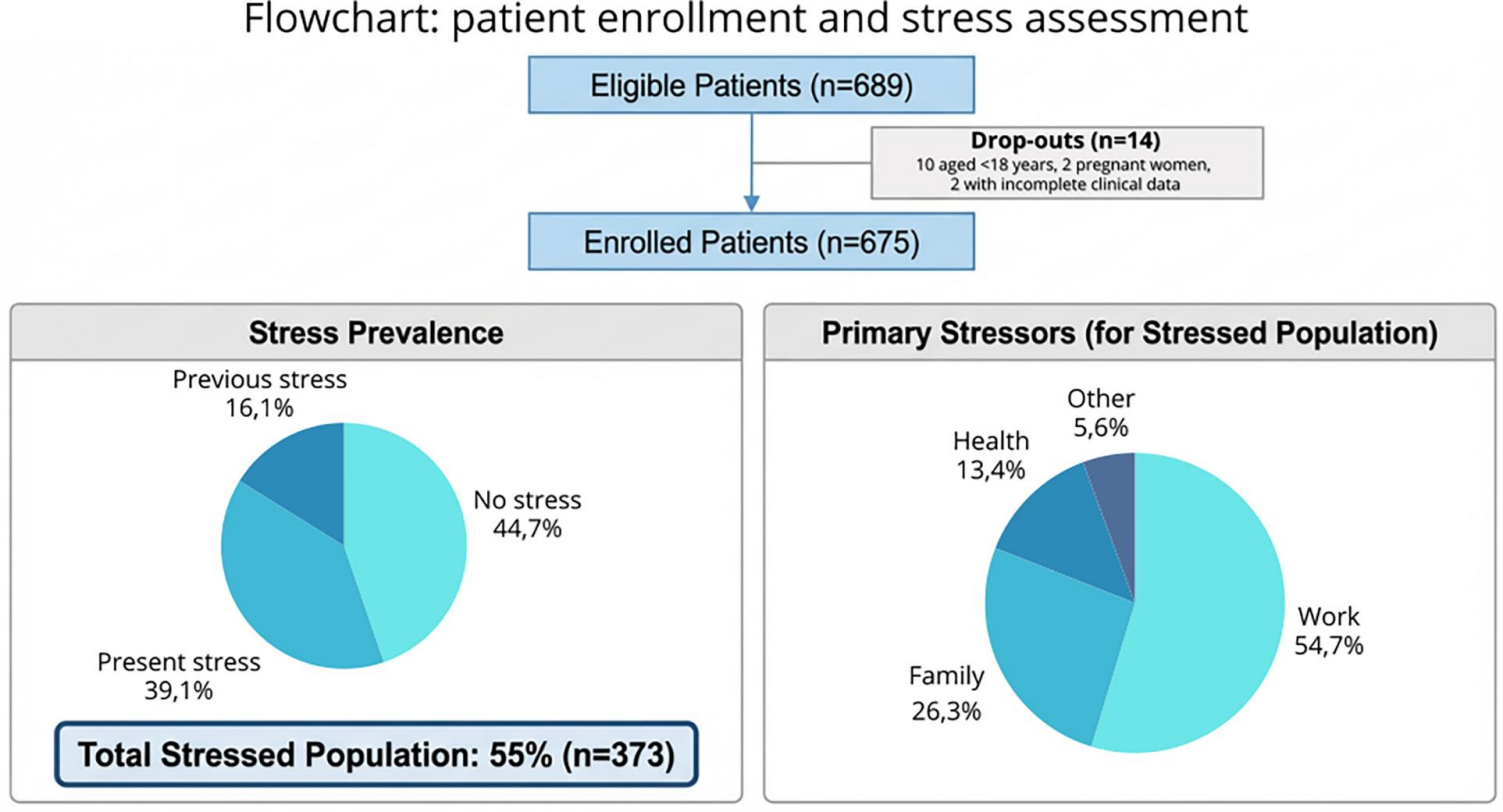
Patient enrollment and stress assessment flowchart. Legend: The flowchart illustrates the recruitment process, from 689 eligible patients to the final study population of 675 enrolled participants after excluding 14 drop-outs. The left pie chart shows the distribution of stress status in the overall population. The right pie chart details the distribution of primary stressors among patients reporting psychological stress.

This multicenter cross-sectional study was conducted in three cardiology outpatient settings in Northern Italy: (1) the cardiology outpatient clinic of Conegliano General Hospital (Treviso, Italy), (2) the Marusia outpatient clinic (Conegliano, Treviso, Italy), and (3) the ABC Study Foundation outpatient clinic in collaboration with a local association (Noi × Noi Association, ETS, Tarzo, Treviso, Italy). This project was named the ABC2X-2026 study. Between 2019 and 2025, 689 consecutive patients were evaluated and considered eligible. Only first visits were included to avoid repeated observations from the same individual. Fourteen patients were excluded from the analysis (10 aged <18 years, 2 pregnant women, and 2 with incomplete clinical data), resulting in a final study sample of 675 patients included in the present statistical analysis (Figure 1).

The study was conducted in agreement with the Declaration of Helsinki and within the institutional framework of the ABC Study Foundation. All data were anonymized before statistical analysis. All participants provided written informed consent for the use of anonymized data for research purposes.

### 2.2 Assessment of cardiovascular parameters and psychological stress

All patients underwent a comprehensive clinical evaluation performed by a cardiologist during the outpatient visit. Data collection included medical history, cardiovascular risk factors, medication use, and lifestyle characteristics.

Blood pressure and heart rate were measured at rest with the patient in the supine position using a validated automated oscillometric BP monitor (Microlife AG, Widnau, Switzerland), according to a standardized protocol consistent with current ESC recommendations for office blood pressure assessment. Patients rested in the supine position for 5 min before the first automated measurement was recorded; the second reading was performed 5 min later, with the patient remaining supine during the interval. Two consecutive measurements were obtained and averaged to determine baseline BP and HR values. Measurements were systematically obtained from the left arm, with the cuff positioned at heart level and the arm relaxed and adequately supported. Appropriate cuff sizes were selected according to arm circumference, including dedicated large cuffs for obese patients when required. Before BP and HR measurement, the absence of recent caffeine intake, smoking, and vigorous physical exercise was specifically checked and verified. In addition, the timing of medication intake before the visit was systematically recorded. All measurements were performed and recorded by a trained nurse before the clinical interview, thereby reducing the possibility that stress classification influenced BP/HR measurement. The physicians conducting the interview were blinded to BP and HR values at the time of stress assessment.

Medical history was systematically reviewed to identify cardiovascular comorbidities. In particular, CAD was defined as documented history of acute coronary syndrome, prior revascularization, or angiographically confirmed significant stenosis. To ensure hemodynamic stability and minimize transient peri-event psychological distress, only patients in a stable clinical phase, at least 6 months after an acute coronary event or revascularization, were included. This criterion allowed a more reliable assessment of the interplay between psychosocial stressors and BP regulation, minimizing the confounding effects of the acute phase.

Psychological stress was assessed through a standardized physician-patient interview. To ensure a uniform understanding of the construct, the cardiologist provided each patient with a formal explanation of stress consistent with the DSM-5 conceptual definition of psychological stress before recording the response (34). Specifically, the physician first asked: “Do you feel stressed?” The term was then clarified as follows: “By stress, we mean a state of psychological tension or distress resulting from external pressures or life events that you feel exceed your ability to cope, potentially affecting your daily functioning or well-being”.

Following the patient's response, the physician conducted a structured interview to characterize the temporal profile of the psychological burden. Stress was categorized into three distinct groups:

1. Present stress, defined as a currently active state of psychological tension that had persisted for at least 3 months before the visit;
2. Previous stress, defined as a psychological burden that had occurred within the previous 5 years but was no longer active, having resolved at least 3 months before the evaluation;
3. No current stress, including patients who reported never having experienced significant stress or whose stress had resolved more than 5 years before enrollment.

The 3-month cut-off was chosen to distinguish ongoing or persistent psychological stress from short-term transient reactions, whereas the 5-year limit was adopted to reduce recall bias and focus on relatively recent stress exposure.

Second, the physician identified the primary stressor by asking the patient to indicate the main cause of their distress. Stressors were categorized as financial/work-related, family/relational, health-related, or other causes (25–29). To ensure procedural consistency, all cardiologists were required to follow the ABC Study Protocol, which specified that the same stress definition had to be provided and that follow-up questions regarding duration and stressors had to be asked in a uniform manner, (Supplementary Table S1).

This structured approach ensured that the patient's self-assessment was anchored to a clinically recognized definition of psychological burden and allowed for a detailed temporal and causal characterization. This methodology, which combines a direct single-item inquiry with a standardized clinical definition and follow-up, is supported by previous studies showing that single-item measures of self-perceived stress have acceptable reliability and validity and may serve as practical tools for identifying psychological distress in clinical research settings (23, 24).

### 2.3 Assessment of potential confounders

Lifestyle habits and potential confounders were assessed during the clinical interview. Smoking status was modeled as a binary variable, defined as current or former smokers vs. non-smokers. Alcohol consumption was evaluated according to local dietary patterns and treated as a binary variable. Participants were classified as drinkers if they reported a daily alcohol intake of at least one standard drink unit. Coffee consumption was also modeled as a binary variable, defined as one or more cups per day vs. no daily consumption.

Physical activity levels were assessed according to the WHO 2020 guidelines (35). Patients were classified as sedentary, defined as no regular physical activity; physically active, defined as <150 min of moderate-intensity or <75 min of vigorous-intensity aerobic activity per week; or sportive, defined as meeting or exceeding these thresholds. Body mass index was entered into the statistical models as a log-transformed variable to account for its non-normal distribution.

To account for the potential long-term impact of the SARS-CoV-2 pandemic, a binary post-COVID enrollment variable was introduced (36, 37). May 4, 2020, the date marking the end of the main national lockdown in Italy, was used as the cut-off to distinguish patients evaluated before the pandemic from those evaluated afterward. No patients were enrolled between March 11 and May 4, 2020, due to the nationwide suspension of non-urgent outpatient clinical activities during the strictest lockdown phase.

### 2.4 Statistical analysis

Continuous variables are presented as mean ± standard deviation (SD), and categorical variables as counts and percentages. Between-group comparisons were performed using ANOVA for continuous variables and the *χ*^2^ test for categorical variables.

Multinomial logistic regression was used to investigate determinants of stress timing and stressors, using no stress as the reference category. Linear regression models were used to evaluate variables associated with SBP, DBP, and HR.

Candidate variables were selected based on clinical relevance and included age, sex, BMI, smoking status, coffee consumption, alcohol consumption, physical activity, hypertension, diabetes mellitus, CAD, post-COVID enrollment, and cardiovascular and anxiolytic treatments. A structured three-step modeling strategy was applied. First, univariable analyses were performed. Second, full multivariable models including all candidate variables were fitted. Third, final parsimonious models were derived by retaining variables according to clinical relevance, potential confounding effect, and statistical contribution. Established clinically relevant covariates were retained regardless of statistical significance, whereas variables without clinical relevance or statistical contribution were removed.

Results from multinomial logistic regressions are reported as relative risk ratios (RRR) with 95% confidence intervals (CI) and *p* values. Results from linear regression models are reported as regression coefficients (β) ± standard error (SE) and *p* values.

Formal interaction terms were introduced into the final parsimonious models as exploratory analyses to evaluate potential effect modification by clinically relevant variables, including age, sex, and CAD. Because these analyses were not adjusted for multiple comparisons, interaction findings were interpreted cautiously and considered hypothesis-generating. When significant interactions were observed, predicted values were derived and graphically displayed. Model assumptions were checked before interpretation. For linear regression models, residual distribution, linearity, homoscedasticity, and collinearity among covariates were assessed. For multinomial logistic regression models, model stability, sparse categories, and collinearity among covariates were evaluated.

All analyses were performed using Stata version 18 (StataCorp LLC, College Station, TX, USA). All tests were two-sided, and a *p*-value < 0.05 was considered statistically significant.

### 2.5 AI disclosures

During the preparation of this manuscript, the authors used generative artificial intelligence (GPAI software) technology exclusively for English editing, grammatical refinement, and improvement of the clarity of the scientific text. The authors explicitly declare that AI tools were not used for data collection, statistical analysis, table preparation, figure generation, or interpretation of the study findings. The final manuscript was thoroughly reviewed and approved by all authors, who retain full responsibility for the integrity, accuracy, and originality of the scientific content of this study.

## 3 Results

Baseline demographic and clinical characteristics according to stress status are presented in Table 1. Overall, 373 (55%) reported psychological stress. Patients reporting present psychological stress were younger and more frequently female compared with those without stress. The prevalence of alcohol consumption and levels of physical activity differed across stress categories. DBP and HR were higher among stressed patients, whereas SBP did not show significant differences. Differences were also observed in medication use (calcium channel blockers and anxiolytics treatment).

**Table 1:** Patients’ baseline characteristics according to stress status.

Variable	Overall population (*n* = 675)	Absence of stress (*n* = 302)	Presence of stress (*n* = 264)	Previous stress (*n* = 109)	*P* value
Demographics and clinical data					
Age at enrolment (years)	65.1 ± 15.0	68.0 ± 15.0	60.1 ± 15.0	69.3 ± 11.7	<0.0001
Females	301 (45%)	113 (37%)	142 (54%)	46 (42%)	<0.0001
Body Mass Index (kg/m^2^)^a^	25.6 ± 4.2	25.9 ± 3.8	25.0 ± 4.4	26.3 ± 4.9	0.01
Current smokers (no/yes)	216 (32%)	87 (28%)	88 (33%)	41 (38%)	0.20
Coffee consumption (no/yes)	548 (81%)	255 (84%)	207 (78%)	86 (79%)	0.15
Alcohol consumption (no/yes)	308 (46%)	152 (50%)	99 (37%)	57 (52%)	0.003
Hypertension (no/yes)	371 (55%)	178 (59%)	123 (47%)	70 (64%)	0.001
Physical exercise (no/yes)	418 (62%)	200 (66%)	149 (56%)	70 (64%)	0.05
Diabetes mellitus (no/yes)	72 (11%)	38 (13%)	19 (7%)	15 (14%)	0.06
Coronary Artery Disease (no/yes)	110 (16%)	56 (19%)	34 (13%)	20 (18%)	0.16
Post Covid Enrolment (no/yes)	545 (81%)	241 (80%)	210 (80%)	94 (86%)	0.58
Cardiovascular characteristics					
Systolic Pressure (mmHg)	147.0 ± 22.1	147.4 ± 22.3	145.0 ± 22.5	150.4 ± 20.3	0.44
Diastolic Pressure (mmHg)	81.8 ± 11.0	80.6 ± 11.4	83.2 ± 10.7	81.6 ± 10.4	0.46
Heart Rate (bpm)	71.4 ± 13.5	70.0 ± 13.9	72.7 ± 13.0	71.8 ± 13.8	0.55
Treatment					
Beta—Blockers (no/yes)	179 (27%)	87 (29%)	59 (22%)	33 (30%)	0.14
Calcium channel blockers (no/yes)	116 (17%)	66 (22%)	32 (12%)	18 (17%)	0.009
ACE inhibitors/ARBs (no/yes)^b^	275 (41%)	134 (44%)	92 (35%)	49 (45%)	0.04
Anxiolytic treatment (no/yes)	105 (16%)	31 (10%)	56 (21%)	18 (17%)	0.002

a Calculated using log-transformed data.

b ACE inhibitors/ARBs, angiotensin—converting enzyme inhibitors/angiotensin II receptor blockers.

### 3.1 Determinants of stress timing

Present stress was reported in 264 (39%) patients, while previous stress in 109 (16%). Multinomial logistic regression analysis showed that younger age, female sex, physical activity and current anxiolytic treatment were independently associated with present stress (Table 2). Interestingly, post-COVID-19 enrolment was found to be associated with previous stress.

**Table 2:** Multinomial logistic regression analysis of variables associated with stress status, using absence of stress as the reference category.

Variable	Univariable models	*P* value	Full multivariable model	*P* value	Parsimonious model	*P* value
	RRR (95% CI)		RRR (95% CI)		RRR (95% CI)	
Present Stress						
Age at enrolment (decades)	0.7 (0.7–0.8)	<0.0001	0.7 (0.6–0.8)	<0.0001	0.6 (0.5–0.7)	<0.0001
Females	1.9 (1.4–2.7)	<0.0001	1.5 (1.0–2.2)	0.05	1.6 (1.1–2.3)	0.01
Physical exercise	0.7 (0.6–0.9)	0.01	0.7 (0.5–0.9)	0.01	0.7 (0.5–0.9)	0.01
Body Mass Index (kg/m^2^)^a^	0.2 (0.1–0.7)	0.007	0.7 (0.2–2.4)	0.56		
Post Covid enrolment	1.0 (0.7–1.5)	0.94	0.9 (0.6–1.6)	0.85		
Hypertension	0.6 (0.4–0.8)	0.003	1.0 (0.6–1.9)	0.91		
Diabetes mellitus	0.5 (0.3–0.9)	0.03	0.8 (0.4–1.6)	0.58		
Coronary Artery Disease	0.6 (0.4–1.0)	0.07	0.9 (0.5–1.5)	0.59		
Coffee consumption	0.7 (0.4–1.0)	0.06	0.8 (0.5–1.4)	0.49		
Alcohol consumption	0.6 (0.4–0.8)	0.002	0.9 (0.6–1.3)	0.64		
Current smokers	1.2 (0.9–1.7)	0.25	1.3 (0.8–1.9)	0.26		
Beta—Blockers	0.7 (0.5–1.0)	0.08	0.9 (0.6–1.5)	0.70		
Calcium channel blockers	0.5 (0.3–0.8)	0.003	0.6 (0.4 −1.1)	0.10		
ACE inhibitors/ARBs^b^	0.7 (0.5–0.9)	0.02	1.1 (0.6–1.9)	0.75		
Anxiolytics treatment	2.4 (1.5–3.8)	<0.0001	2.4 (1.4–4.0)	0.002	2.4 (1.4–4.0)	0.001
Previous stress						
Age at enrolment (decades)	1.1 (0.9–1.2)	0.60	1.0 (0.8–1.2)	0.90		
Females	1.2 (0.8–1.9)	0.38	1.3 (0.8–2.2)	0.31		
Physical exercise	0.8 (0.6–1.1)	0.22	1.0 (0.7–1.4)	0.97		
Body Mass Index (kg/m^2^)^a^	1.6 (0.4–6.1)	0.51	1.6 (0.3–7.8)	0.58		
Post Covid enrolment	1.6 (0.9–2.9)	0.14	2.8 (1.4–5.4)	0.003	2.6 (1.4–5.1)	0.004
Hypertension	1.3 (0.8–2.0)	0.34	1.6 (0.8–3.2)	0.21		
Diabetes mellitus	1.1 (0.6–2.1)	0.75	1.1 (0.5–2.2)	0.83		
Coronary Artery Disease	1.0 (0.6–1.7)	0.96	1.0 (0.5–1.9)	0.90		
Coffee consumption	0.7 (0.4–1.2)	0.19	0.8 (0.5–1.5)	0.57		
Alcohol consumption	1.1 (0.7–1.7)	0.73	1.3 (0.8–2.1)	0.33		
Current smokers	1.5 (0.9–2.4)	0.09	1.3 (0.8–2.2)	0.30		
Beta—Blockers	1.1 (0.7–1.7)	0.8	0.8 (0.5–1.5)	0.56		
Calcium channel blockers	0.7 (0.4–1.3)	0.24	0.5 (0.3–1.0)	0.05		
ACE inhibitors/ARBs^b^	1.0 (0.7–1.6)	0.92	0.7 (0.4–1.3)	0.30		
Anxiolytics treatment	1.7 (0.9–3.2)	0.09	1.4 (0.7–2.7)	0.36		

RRR, relative risk ratio.

a Calculated using log-transformed data.

b ACE inhibitors/ARBs, angiotensin-converting enzyme inhibitors/angiotensin II receptor blockers.

**Figure 2:**
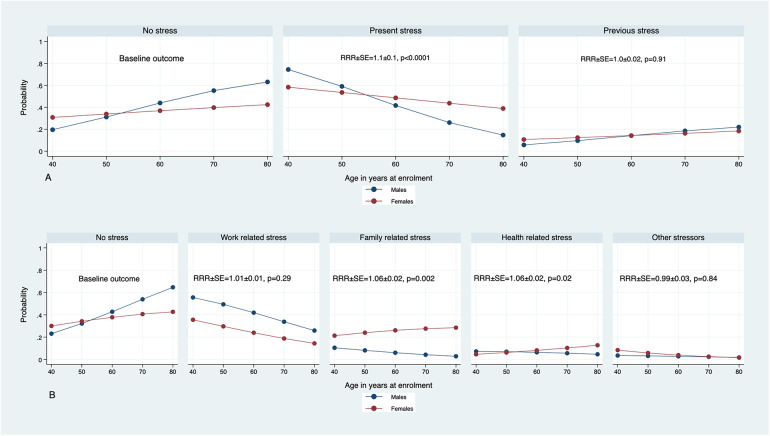
Exploratory multinomial logistic regression analyses of predicted probabilities of stress status and primary stressors according to age and sex. Legend: Panel **(A)** shows exploratory predicted probabilities of stress status, including no stress, present stress, and previous stress, according to age and sex. Panel **(B)** shows exploratory predicted probabilities of primary stressor categories, including financial/work-related, family/relational, health-related, and other stressors, according to age and sex. Predicted probabilities were derived from the final multinomial logistic regression models. Interaction analyses were exploratory and not adjusted for multiple comparisons; therefore, these findings should be interpreted as hypothesis-generating.

Interaction between age and sex is shown in Figure 2 (Panel A). The probability of reporting no stress increased with age among males, whereas it showed a more gradual increase among females. Conversely, the probability of present stress decreased progressively with age in both sexes, with a steeper decline observed among males. Among females, the reduction in present stress probability across age groups was less pronounced.

### 3.2 Sources of stress

Primary stressors were classified as financial/work-related, family/relational, health-related, or other causes (Table 3). Among patients who reported psychological stress, financial/work-related stress was the predominant category, reported by 120 patients (55%), followed by family/relational stress in 98 patients (26%), health-related stress in 50 patients (13%), and other causes in 21 patients (6%).

**Table 3:** Multinomial logistic regression analysis of variables associated with primary stressors, using absence of stress as the reference category^a^.

Variable	Full multivariable model		Parsimonious model	
	RRR^a^ (95% CI)	*P* value	RRR (95% CI)	*P* value
Financial/Work related stress				
Age at enrolment (decades)	0.7 (0.6–0.8)	<0.0001	0.7 (0.6–0.8)	<0.0001
Females	0.7 (0.5–1.2)	0.24		
Physical exercise	0.8 (0.6–1.1)	0.20		
Family/relational related stress				
Age at enrolment (decades)	0.8 (0.7–1.0)	0.71	0.8 (0.7–0.9)	0.03
Females	5.6 (3.0–10.2)	<0.0001	6.8 (3.9–11.9)	<0.0001
Physical exercise	0.7 (0.4–0.9)	0.04	0.7 (0.5–0.9)	0.03
Health related stress				
Age at enrolment (decades)	1.0 (0.8–1.3)	0.94		
Females	2.1 (1.1–4.3)	0.03	2.2 (1.2–4.3)	0.01
Physical exercise	0.6 (0.4–1.0)	0.06	0.6 (0.3–0.9)	0.03
Other stressors				
Age at enrolment (decades)	0.5 (0.3–0.7)	<0.0001	0.6 (0.5–0.8)	<0.0001
Females	2.8 (1.0–8.1)	0.05		
Physical exercise	0.6 (0.3–1.2)	0.16		

RRR, relative risk ratio; CI, confidence intervals.

a The models also included the following variables: body mass index, coffee consumption, alcohol consumption, smoke, diabetes mellitus, CAD, hypertension, post-COVID enrolment.

Younger age was independently associated with financial/work-related, family/relational related and other causes of stress. Female sex was independently associated with disease-related stress and with family/relational related stress. Physical exercise was inversely associated with family/relational related and health-related stress. Predicted probabilities of interaction between age and sex are shown in Figure 2 (Panel B). With increasing age, female patients reported higher family/relational related and health-related stress, whereas they declined in men over time. No significant interactions were found for work or other stress factors.

### 3.3 Association between stress and cardiovascular parameters

Parsimonious multivariable linear regression models showed that age was positively independently associated with higher SBP, male sex with higher DBP, physical activity with reduced HR and CAD with lower DBP and HR (Table 4). Present stress was independently associated with higher DBP, whereas previous stress was not. Neither present nor previous stress results associated with SBP or HR. Parsimonious models also included the following variables: BMI, Diabetes Mellitus, ACE inhibitors or angiotensin II receptor blockers (ARBs), Beta-Blockers.

**Table 4:** Parsimonious multivariable linear regression models for systolic and diastolic blood pressure, and heart rate^a^.

Variable	Systolic blood pressure		Diastolic blood pressure		Heart rate	
	β ± S.E.	*P* value	β ± S.E.	*P* value	β ± S.E.	*P* value
Age at enrolment (decades)	4.5 ± 0.6	<0.0001	0.2 ± 0.3	0.54	0.2 ± 0.4	0.61
Females	−2.6 ± 1.6	0.12	−4.0 ± 0.8	<0.0001	1.6 ± 1.1	0.13
Present stress	2.5 ± 1.8	0.17	3.0 ± 0.9	0.001	1.4 ± 1.1	0.22
Previous stress	1.7 ± 2.3	0.45	1.9 ± 1.1	0.10	0.1 ± 1.4	0.95
Physical exercise	0.3 ± 1.2	0.81	0.4 ± 0.6	0.52	−2.7 ± 0.8	<0.0001
Coronary Artery Disease	−3.8 ± 2.2	0.09	−2.6 ± 1.1	0.02	−5.8 ± 1.5	<0.0001

SE, standard error.

a The parsimonious models included even the following variables: body mass index, diabetes mellitus, ACE inhibitors/angiotensin II receptor blockers, Beta—Blockers (not displayed in the table).

Calcium channel blocker use was considered as a candidate covariate because it differed between stress groups and may influence DBP. However, it was not significantly associated with DBP in linear regression analysis (β = −1.05 ± 1.13 mmHg, *p* = 0.353) and was therefore not retained in the final parsimonious DBP model.

### 3.4 Diastolic blood pressure and stress status

In the overall sample, DBP resulted higher among patients with present stress and not among patients with previous stress as compared to non-stressed patients. DBP differences were also observed among males, but not among females. Similarly, among patients with CAD, DBP differed according to stress status, whereas no significant differences were observed among patients without CAD (Supplementary Table S2).

### 3.5 Exploratory interaction analyses

Exploratory interaction analyses suggested that the association between present stress and DBP varied according to age and CAD status.

**Figure 3:**
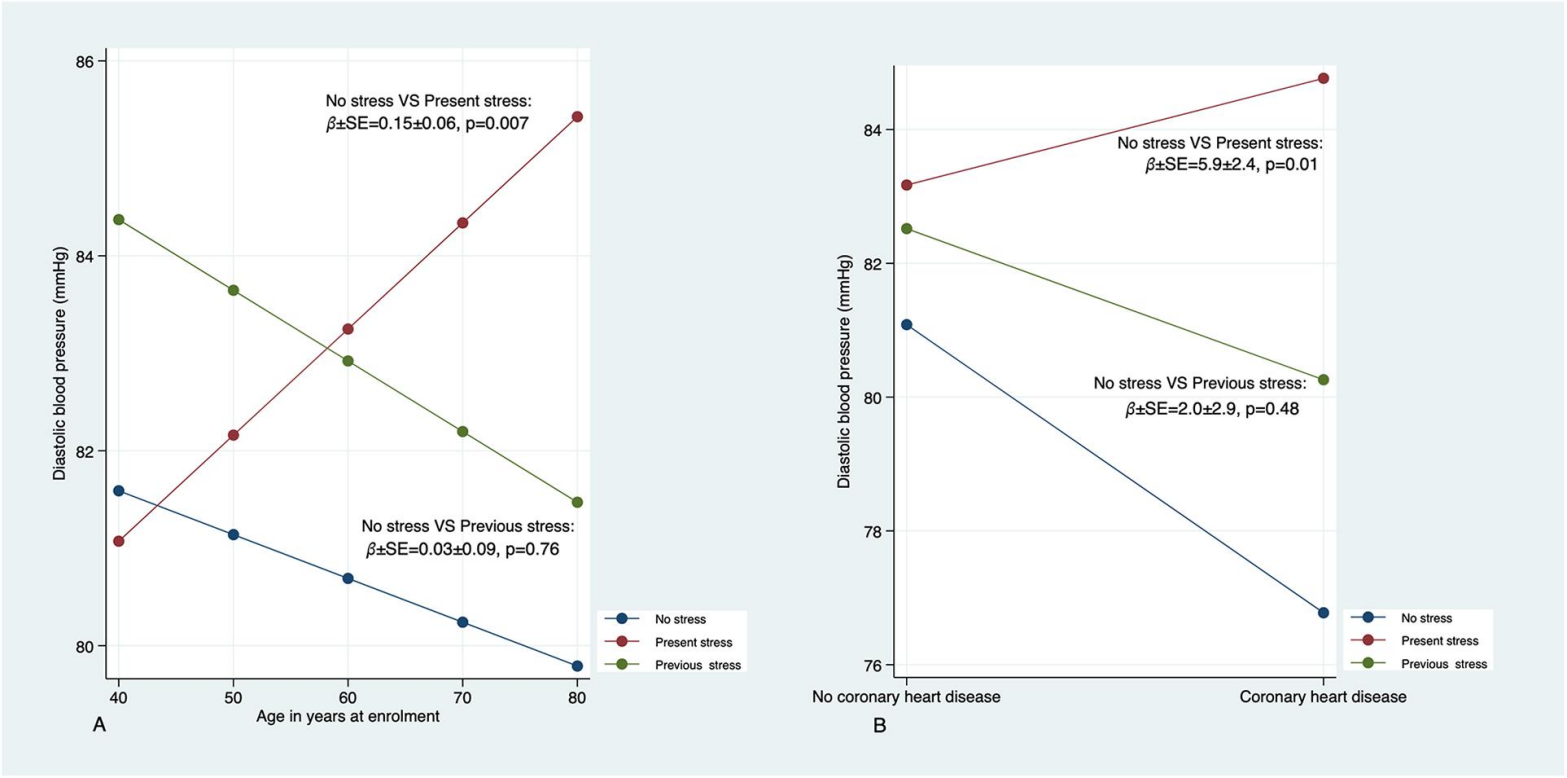
Exploratory interaction analyses evaluating predicted diastolic blood pressure according to stress status, age, and coronary artery disease. Legend: Panel **(A)** shows exploratory predicted DBP values according to age and stress status. Panel **(B)** shows exploratory predicted DBP values according to CAD status and stress status. Predicted values were derived from the final multivariable linear regression models. Interaction analyses were exploratory and not adjusted for multiple comparisons; therefore, these findings should be interpreted as hypothesis-generating.

Predicted DBP values according to age and stress category are shown in Figure 3, Panel A. Among individuals without stress, DBP tended to decline with increasing age. In contrast, among patients reporting present stress, predicted DBP increased progressively with age. No clear age-related interaction pattern was observed among patients with previous stress.

As shown in Figure 3, Panel B, the association between present stress and DBP appeared more pronounced among patients with established CAD than among those without CAD. Conversely, no relevant interaction was observed between previous stress and CAD status.

## 4 Discussion

In this multicenter cross-sectional study of cardiology outpatients, we observed an overall stress prevalence of 55% (*n* = 373). This finding appears consistent with global epidemiological data from the INTERHEART study (22). Yet, the present analysis reveals that present and previous stress are associated with two distinct clinical entities characterized by different demographic and temporal profiles. Present stress resulted independently associated with female sex, younger age, physical inactivity and anxiolytic treatment (16, 22, 26, 37). This association suggests the presence of a sub-group of patients more likely to report psychological stress. In contrast, previous stress appeared to reflect a transient collective experience related to the COVID-19 pandemic, as it was predominantly reported among patients evaluated after the end of the national lockdown on May 4, 2020 (36, 37). This divergence was further supported by the interaction models showing distinct age- and sex-related patterns: while previous stress showed no marked sex-specific divergence, present stress exhibited a significant age–sex interaction. The probability of reporting present stress declined markedly with age in men, whereas women remained persistently prone to reporting stress across all age groups. These results highlight a specific clinical pattern: not only are women more likely to report stress than men, but while the prevalence of stress is lower in older age categories among men, this difference is not observed among women.

Furthermore, analysis of stress sources showed that differences extend even to the stressor's type. Financial/work-related stress was primarily associated with younger age categories, while significant age-sex interactions were observed for family- and health-related stressors. Among men, the probabilities of reporting these stressors were lower in older groups, while among women they were not.

### 4.1 Present vs. previous stress

The results of this study suggest that present and previous stress represent distinct clinical states with different hemodynamic profiles. Even when adjusting the models, present stress remained independently associated with higher DBP, with values approximately 3 mmHg higher than those observed in individuals without stress. This association remained robust in both parsimonious and fully adjusted models, which accounted for a comprehensive set of potential confounders—including demographics, lifestyle habits, and clinical comorbidities such as CAD and diabetes. The association persisted even after adjusting for all cardiovascular medications. This suggests that the observed DBP difference is independently linked to present psychological stress rather than being secondary to pharmacological treatment or other clinical variables. According to the meta-analysis by Lewington (2002), even small differences in usual blood pressure are strongly associated with vascular mortality; for instance, a 5 mmHg difference in usual DBP is associated with approximately a 40% lower risk of stroke death and a 30% lower risk of death from ischemic heart disease (38). Therefore, the 3 mmHg elevation observed in our “present stress” group may represent a clinically non-negligible factor for long-term cardiovascular risk. In contrast, previous stress was not significantly associated with DBP after adjustment for confounding variables.

However, analysis of variance revealed a clinically relevant intermediate pattern. Patients reporting previous stress exhibited DBP values numerically between those with present stress and those without stress. This association may reflect an “hemodynamic scar” effect, characterized by higher DBP levels in individuals with a history of stress compared to those who never experienced it. This observation is consistent with McEwen's theory of allostatic load (6, 7), which describes the physiological burden associated with chronic exposure to stress. According to this model, the cardiovascular system may not immediately reset following stress resolution. It could be hypothesized that prolonged activation of the stress response involving the hypothalamic-pituitary-adrenal axis regulation, chronic inflammation, and endothelial dysfunction, could explain the intermediate DBP values observed in the previous-stress group (10, 11).

### 4.2 Stress and diastolic blood pressure

An independent positive association between present stress and DBP levels was observed, whereas no such associations were found for SBP or HR. Although the present study precludes drawing any causal inferences, a potential mechanistic explanation is provided by other studies, which suggest that psychological stress is associated with the activation of the sympathetic nervous system, thereby modifying cardiovascular responses (6, 9). These different patterns may be interpreted in light of the distinct hemodynamic determinants of SBP, DBP, and HR.

### 4.3 Interaction with age and coronary artery disease

In the present study, a significant interaction between age and present stress on DBP was observed. Among individuals without stress, DBP was lower in older age groups compared with younger ones; conversely, among those reporting stress, DBP was higher in the older sub-groups. Even if these findings do not allow for causal inference, a possible interpretation could consider factors such as structural alterations, endothelial dysfunction, or reduced baroreflex sensitivity, which may amplify the hemodynamic impact of psychological stress (30, 31). In this light, age could be considered a potential effect modifier in the association between stress and DBP within this cross-sectional observation.

Furthermore, the association between present stress and DBP was more pronounced among patients with established CAD than among those without CAD. Although our cross-sectional data do not allow causal inference, this finding may suggest a differential hemodynamic profile associated with present stress according to underlying cardiovascular status. In patients with CAD, structural and functional vascular alterations related to atherosclerotic disease may increase susceptibility to sympathetic activation and peripheral vasoconstriction during psychological distress, thereby making the association between stress and DBP more evident (1, 3, 32, 33). However, these interaction analyses, particularly for CAD, should be interpreted cautiously. The relatively limited number of patients with CAD, the cross-sectional design, and the possibility of residual confounding prevent definitive conclusions regarding effect modification. This finding should be considered exploratory and hypothesis-generating. Nevertheless, it may identify patients with established CAD as a potentially vulnerable subgroup in whom ongoing psychological stress is associated with a more pronounced DBP response (32). Further prospective studies with larger CAD populations and repeated BP and stress assessments are needed to confirm this observation.

### 4.4 Clinical implications

The present findings may support the routine assessment of psychological stress during cardiology outpatient visits (3). A simple structured clinical interview was able to identify stress patterns associated with measurable hemodynamic differences. Yet, distinguishing between present and previous stress may improve clinical interpretation and help identify patients who could benefit from targeted behavioral or psychosocial interventions. In particular, older individuals and patients with established CAD may represent subgroups with increased vulnerability to stress-related vascular effects.

### 4.5 Strengths and limitations

The strengths of this study include the real-world cardiology outpatient setting, the consecutive enrollment of patients undergoing first visits, and the integration of stress timing and stress source assessment within a structured clinical framework.

The present study has several limitations that should be considered when interpreting the results. First, psychological stress was assessed through a direct, physician-guided interview rather than using multi-item psychometric scales. While this might be seen as a limitation in capturing the multidimensionality of stress, we sought to maximize the reliability of the assessment through a rigorous standardization process. All participating cardiologists were trained in the ABC Study protocol, ensuring that a formal definition of stress based on DSM-5 criteria was read to each patient. This approach ensured that the “Presence of stress/Not presence of stress” response was anchored to a clinically recognized construct. As demonstrated by Littman et al. and Elo et al., single-item measures are validated proxies for psychological distress in clinical settings, offering significant ecological validity (23, 24).

Second, the assessment of “previous stress” (occurring between 3 months and 5 years prior) is inherently susceptible to recall bias and potential misclassification. Patients may have difficulty accurately recalling the exact timing or intensity of past stressors, and their current emotional or physical state might influence their perception of past events. This retrospective self-reporting could lead to an over- or underestimation of the association between past stress and current blood pressure levels. Therefore, findings related to previous stress should be interpreted cautiously and require confirmation in prospective studies using repeated stress assessments.

Yet, while home patient's treatment was accurately recorded, we did not systematically collect data regarding medication timing and long-term treatment adherence.

Additionally, BP was assessed via office readings, which limits our ability to track short-term variability or fully rule out a “white-coat effect”.

Moreover, the clinical interpretation of the approximately 3 mmHg higher DBP observed in patients with present stress should be cautious. Although this difference may be relevant at a population level, particularly in cardiology outpatients, the cross-sectional design of the present study does not allow causal inference or assessment of whether this BP difference persists over time. BP was measured during a single outpatient visit, and therefore transient situational influences cannot be fully excluded. Consequently, our findings should be interpreted as an association between ongoing psychological stress and higher DBP at the time of clinical evaluation, rather than evidence of a sustained hemodynamic effect. Prospective studies with repeated stress and BP assessments are needed to determine whether this difference is persistent and clinically prognostic.

Also, the interaction analyses should be considered exploratory and hypothesis-generating, particularly for CAD, given the relatively limited number of patients with established disease and the absence of correction for multiple testing. Therefore, these findings require confirmation in larger prospective cohorts.

Finally, the present study was conducted in cardiology outpatient clinics in Northern Italy; therefore, the findings mainly reflect this regional demographic, sociocultural, and healthcare context. Generalizability to other ethnic groups, cardiovascular risk profiles, psychosocial backgrounds, and healthcare systems may be limited. Further studies in more diverse populations are needed to confirm these findings.

## 5 Conclusions

In this multicenter cohort of cardiology outpatients, psychological stress was highly prevalent and associated with specific hemodynamic patterns, particularly for DBP. Present and previous stress appear to represent distinct clinical states with different cardiovascular implications. Exploratory interaction analyses suggested that older individuals and patients with established CAD may represent subgroups with greater susceptibility to stress-related DBP differences.

These findings suggest that systematic evaluation of psychological stress in routine cardiology practice may help to identify stress-related hemodynamic differences in this outpatient setting.

## Data Availability

The raw data supporting the conclusions of this article will be made available by the authors, without undue reservation.
