# Supplementary material for: Psychological stress and diastolic blood pressure in cardiology outpatients: a multicenter cross-sectional study (from the ABC2X-2026 study)
Source: Front Cardiovasc Med. 2026 Jun 17;13:1865898. doi: 10.3389/fcvm.2026.1865898 (PMC13318798; doi:10.3389/fcvm.2026.1865898)
Supplement: Supplementary file 1 [file Table1.docx]

Supplementary Table 1. Psychological stress interview and classification algorithm

| Assessment step | Interview procedure | Final classification |
| --- | --- | --- |
| Initial screening | The physician asked: “Do you feel stressed?” The term stress was explained using a standardized definition: psychological tension or distress caused by external pressures or life events perceived as exceeding coping capacity and potentially affecting daily functioning or well-being. | Stress present or absent according to patient self-report after standardized explanation. |
| Timing of stress | Patients reporting stress were asked whether the stress was active, persistent, or resolved, and when it had occurred. | Present stress, previous stress, or no stress. |
| Present stress | Stress active at the time of evaluation and persistent for at least 3 months before the visit. | Classified as present stress. |
| Previous stress | Stress occurring within the previous 5 years but no longer active and resolved for at least 3 months before evaluation. | Classified as previous stress. |
| No stress | No significant stress reported, or stress resolved more than 5 years before the visit. | Classified as no stress. |
| Primary stressor | Patients with present or previous stress were asked to indicate the main cause of distress. | Classified as financial /work-related, family/relational, health-related, or other. |
| Procedural standardization | All cardiologists followed the ABC Study protocol. BP and HR were measured by a trained nurse before the interview, and physicians were blinded to these values during stress assessment. | Standardized interview procedure across centers. |
