# Supplementary material for: Psychological stress and diastolic blood pressure in cardiology outpatients: a multicenter cross-sectional study (from the ABC2X-2026 study)
Source: Front Cardiovasc Med. 2026 Jun 17;13:1865898. doi: 10.3389/fcvm.2026.1865898 (PMC13318798; doi:10.3389/fcvm.2026.1865898)
Supplement: Supplementary file 2 [file Table2.docx]

Supplementary Table 2. Diastolic blood pressure according to stress status in the overall population and selected subgroups: ANOVA analysis

| **Diastolic blood pressure Values** | **No stress** | **Present stress** | **Previous stress** | **F value** | **P value** |
| --- | --- | --- | --- | --- | --- |
|  | **Mean ± S.D.** | **Mean ± S.D.** | **Mean ± S.D.** |  |  |
| Overall | 80.6 ± 11.4* | 83.2 ± 10.7* | 81.6 ± 10.5 | 3.94 | 0.02 |
| Male patients | 81.4 ± 11.1* | 86.0 ± 10.8* | 83.3 ± 9.5 | 6.98 | 0.001 |
| Female patients | 79.3 ± 11.7 | 80.8 ± 10.0 | 79.3 ± 11.5 | 0.64 | 0.53 |
| Patients without CAD | 81.9 ± 11.1 | 83.2 ± 10.6 | 82.4 ± 10.3 | 0.80 | 0.45 |
| Patients with CAD | 74.9 ± 10.7* | 83.5 ± 11.7* | 78.3 ± 10.8 | 6.41 | 0.002 |

Significant pairwise difference between groups.
CAD = coronary artery disease; SD = standard deviation.
